# The Aryl Hydrocarbon Receptor and Tumor Immunity

**DOI:** 10.3389/fimmu.2018.00286

**Published:** 2018-02-13

**Authors:** Ping Xue, Jinrong Fu, Yufeng Zhou

**Affiliations:** ^1^Children’s Hospital and Institute of Biomedical Sciences, Fudan University, Shanghai, China; ^2^Key Laboratory of Neonatal Diseases, Ministry of Health, Shanghai, China

**Keywords:** *aryl hydrocarbon receptor*, tumor immunity, tumor development, immune surveillance, cancer immunotherapy

## Abstract

The aryl hydrocarbon receptor (AhR) is an important cytosolic, ligand-dependent transcription factor. Emerging evidence suggests the promoting role of the AhR in the initiation, promotion, progression, invasion, and metastasis of cancer cells. Studies on various tumor types and tumor cell lines have shown high AhR expression, suggesting that AhR is activated constitutively in tumors and facilitates their growth. Interestingly, immune evasion has been recognized as an emerging hallmark feature of cancer. A connection between the AhR and immune system has been recognized, which has been suggested as an immunosuppressive effector on different types of immune cells. Certain cancers can escape immune recognition *via* AhR signaling pathways. This review discusses the role of the AhR in tumor immunity and its potential mechanism of action in the tumor microenvironment.

## Introduction

In 2011, Hanahan and Weinberg proposed eight hallmarks of cancer: self-sufficiency in growth signals; blockade of antigrowth signals; limitless replicative potency; sustained angiogenesis; anti-apoptosis; metabolic reprogramming; tumor infiltration and metastasis; and evasion of the immune system (1). Increasing evidence suggests that the development and progression of cancer cells result from a cancer-induced immunosuppressive situation, one that the immune system cannot recognize. “Immune evasion” is an emerging hallmark feature of cancer (2).

The aryl hydrocarbon receptor (AhR) is an important cytosolic, ligand-activated receptor expressed in various mammals (3, 4). This receptor was studied first as a receptor to the exogenous ligand 2,3,7,8-tetrachlorodibenzo-*p*-dioxin (TCDD) (5). A connection between the AhR and the immune system had been recognized through a pathway reacting with TCDD, which had been reported to be an immunosuppressive effector on T cells and dendritic cells (DCs) in animals and humans (6).

In 2005, Funatake and colleagues hypothesized that regulatory T cells can be generated through an AhR-dependent mechanism (7). Some studies showed that AhR-deficient mice are prone to autoimmunity (8, 9), whereas AhR-responsive mouse strains with constitutive expression of the AhR have been shown to be more susceptible to developing malignancies (10). Some studies suggested that several types of human cancer cells showed higher numbers of copies of the AhR than normal cells (11). The potential function of the AhR in carcinogenesis in different types of cancer has been explored for several years (12, 13). The AhR may affect the proliferation, tissue invasion, metastasis, and angiogenesis of cancer cells. In addition, certain cancer types can escape from immune recognition *via* an AhR pathway, as shown in malignant gliomas by Opitz and colleagues (14). A tumor-promoting role of the AhR as well as its function in the immune system have been recognized. However, studies on the role of the AhR in tumor immunity are scarce.

Here, we present a brief overview of recent investigations on the role of the AhR and potential mechanism of action (MoA) in tumor immunity. We hope our review serves as a “roadmap” to guide future studies and even future therapeutic perspectives for malignancies.

## Background of the AhR

### Fundamental Information of the AhR

The AhR belongs to basic helix–loop–helix/Per-ARNT-Sim (bHLH-PAS) transcription factor families (5). Poland and Knutson stated that TCDD, benzo(a)pyrene, and polycyclic aromatic hydrocarbons (PAHs) exert their biologic actions by binding directly to the AhR, a cytosolic receptor (15). The AhR is a unique member of the bHLH-PAS family known to be in an activated state by integrating with exogenous or endogenous ligands (16, 17).

**Figure 1:**
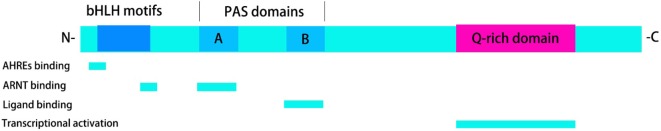
Functional structure of the aryl hydrocarbon receptor (AhR). The functional structure of the AhR protein consists of three parts: the basic helix–loop–helix (bHLH) motifs, the Per-ARNT-Sim (PAS) domains, and a Q-rich domain. bHLH motifs are involved in the activity of aryl hydrocarbon response elements (AHREs) binding and AhR nuclear translocator (ARNT) binding. PAS domains are required for ARNT binding and ligand binding. Transcriptional activation can be observed in Q-rich domain.

The functional structure of the AhR protein comprises three parts: the bHLH motif, the PAS domains, and a Q-rich domain. The basic domain of the bHLH motif is located at the N-terminal region of the AhR protein. The latter binds the AhR to the promoter region of target genes at consistent regulatory sequences termed “aryl hydrocarbon response elements” (AHREs), as well as at dioxin-response elements (DREs). The PAS domains help the formation of a heterozygous protein complex by connecting with the AhR nuclear translocator (ARNT) and binding with the ligand. At the C-terminal region of the protein is a Q-rich domain that affects the recruitment and transcriptional activation of the motif (Figure 1).

**Figure 2:**
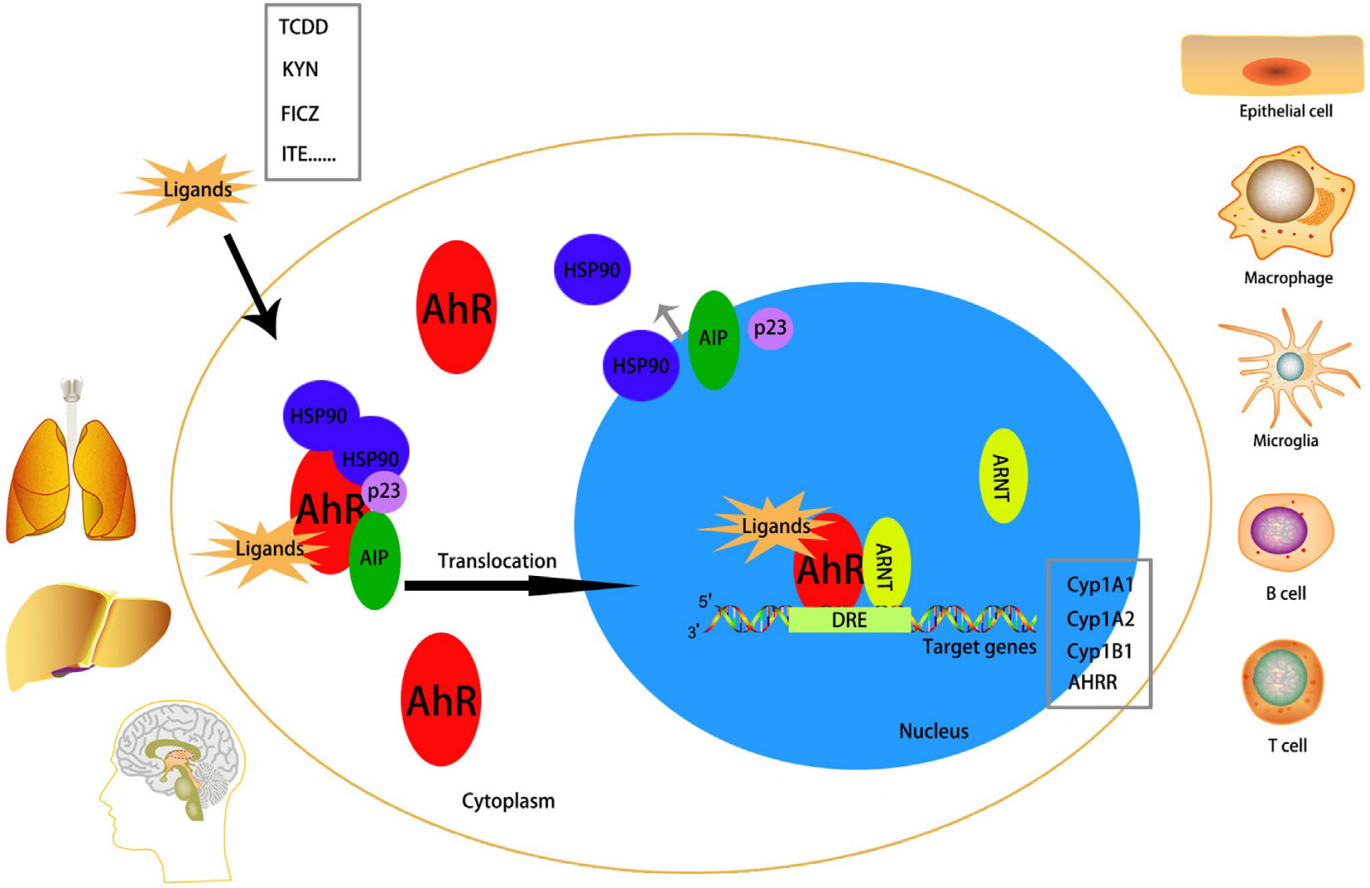
Mechanism of activation of the aryl hydrocarbon receptor (AhR). The AhR is abundantly expressed in lung, liver, and brain. It can be activated in many cell types, including epithelial cell, microglia, macrophage, B cell, T cell, etc. Without a ligand, AhR is inactivated in the cytoplasm as a part of a complex with heat shock protein (HSP)90, AhR-interacting protein (AIP), and p23. After binding with an exo/endogenous ligand, the AhR will be activated and translocates to the nucleus to interact with AhR nuclear translocator (ARNT) and simultaneously detaches from the complex. The AhR/ARNT heterodimer finally binds to the dioxin-response elements (DREs), which is called the promoter region of target genes [classical target genes include cytochrome P450 (Cyp)1a1, Cyp1a2, Cyp1b1, and AHRR], to promote transcriptional activation.

In the absence of ligands, the AhR is located in the cytoplasm as one part of a protein complex comprising heat shock protein 90, p23, and AhR-interacting protein (18–20). Upon binding to ligands such as TCDD, 6-formylindolo[3,2-b]carbazole (FICZ), kynurenine, or 2-(1′H-indole-3′-carbonyl)-thiazole-4-carboxylic acid methyl ester (ITE), the AhR complex is activated. This action is followed by translocation to the nucleus, release from chaperone proteins, and interaction with ARNT. The chaperone proteins can protect the AhR from proteolysis and retain a propitious construction for ligand binding (21). The AhR–ARNT heterodimer correlates with signaling factors (e.g., chromatin remodeling factors, histone acetyltransferases, and transcriptional factors) and finally binds to DREs or AHREs to promote transcriptional regulation (22, 23). Classical AhR target genes include cytochrome P450 (Cyp)1a1, Cyp1a2, Cyp1b1, and AhR repressor (Figure 2).

The AhR is distributed in almost all tissues in humans and expressed abundantly in the placenta, liver, and lungs (24, 25). The AhR can be activated in epithelial cells, Langerhans cells, microglias, T cells, B cells, natural killer (NK) cells, DCs, and macrophages (26–32).

### AhR Ligands

The AhR is activated or inhibited by various types of exogenous and endogenous ligands that bind to it. Different types of ligand interactions with the AhR protein result in different effects (33).

#### Exogenous/Xenobiotic Ligands

The best-characterized high-affinity exogenous/xenobiotic ligands for the AhR are environmental contaminants such as halogenated aromatic hydrocarbons, polychlorinated biphenyls, and PAHs. A well-known prototypic exogenous ligand for the AhR is TCDD, an environmental pollutant with high toxicity. TCDD is a specific epigenetic carcinogen and a potential tumor promoter (12, 34). Exposure to TCDD can produce diverse specific toxic (immunotoxicity, hepatotoxicity, tumor promotion, cardiotoxicity, reproductive toxicity, dermal toxicity, teratogenesis, wasting syndrome, lethality, and endocrine disruption) and biologic effects (35). A rich body of evidence (*in vivo* and *in vitro*) supports these phenomena. AhR^(−/−)^ mice are not sensitive to the toxic activities of TCDD or TCDD-like toxicants (36–38).

#### Endogenous Ligands

It is reasonable to suspect that endogenous ligands must exist for the AhR because it can be activated in some cell types without an exogenous ligand being present (39). Different types of endogenous ligands have been isolated from mammalian tissues, such as indigo and indirubin from human urinary products (40), ITE from the lungs (39), kynurenine and kynurenic acid from the brain (41), and others such as equilenin, arachidonic acid metabolites, and FICZ (42).

Almost all of the endogenous/natural ligands that depend on DRE have been proposed to be AhR agonists. Indigo and indirubin compete for receptor occupancy with TCDD and upregulate the activity of Cyp1a1 monooxygenase in human hepatoma cell lines and in rodent models (43, 44). Equilenin is an estrogen produced by pregnant mares and has been recognized as an AhR agonist. Equilenin has been studied in human HepG2 cells; a half-maximal response (EC_50_) of 30 µM of equilenin can produce a considerable increase in expression of Cyp1a1 mRNA and DRE-mediated reporter activity (45). Potential endogenous AhR ligands from metabolites of arachidonic acid include lipoxin A4 and prostaglandins (PGs). AhR activation by lipoxin A4 induces expression of Cyp1a1 and Cyp1a2 monooxygenases, and lipoxin A4 also serves as a substrate of these enzymes. This phenomenon has been shown in mouse hepatoma cells (46). Furthermore, PG-G2 function has been examined in a murine hepatoma cell line by dose–response assays, indicating that it can induce DRE-dependent transcription with a higher EC_50_ than that elicited by lipoxin A4. PG-G2 may be a weak ligand of the AhR (47). Moreover, heme metabolites could be candidate endogenous ligands for the AhR, of which bilirubin has been suggested to be the most important. Sinal and Bend demonstrated that the gene expression and enzymatic activity of Cyp1a1 can be modulated directly by bilirubin *via* an AhR pathway in mouse hepatoma cells (48). Among the endogenous ligands mentioned earlier, ITE and kynurenine have garnered more attention from immunologists and oncologists in recent years.

##### 2 -(1′H-Indole-3′-Carbonyl)-Thiazole-4-Carboxylic Acid Methyl Ester

2-(1′H-Indole-3′-carbonyl)-thiazole-4-carboxylic acid methyl ester (ITE) was extracted from porcine lung tissues and shown to be an agonist of the AhR by Song and colleagues in 2002 (39). They extracted and purified ITE by ultraviolet spectroscopy, electron-impact mass spectrometry, Fourier-transform infrared spectroscopy, and proton nuclear magnetic resonance spectroscopy. Upon exposure to ITE, the AhR binds to the DRE domain and induces increased expression of Cyp1a1 mRNA and DRE-dependent reporter activity, showing that ITE is a ligand and agonist of the AhR. Competitive-binding studies and experiments based on sucrose gradient sedimentation have suggested that ITE competes with TCDD for binding with the AhR from human, murine, killifish, and zebrafish. Its binding affinity (*K*_i_) to the AhR (3 nM) has been shown to be slightly lower than that of the classical AhR ligand TCDD (*K*_i_ = 0.5 nM).

The biologic function of ITE in the immune system has been studied. Quintana and colleagues showed that the progression of experimental autoimmune encephalomyelitis (EAE) is inhibited effectively by ITE treatment *in vivo* and that ITE acts on DCs and T cells through binding with the AhR (49). Nugent LF et al. also studied ITE for its capacity to suppress the development of experimental autoimmune uveitis (EAU) and relevant immune responses. They showed that ITE can suppress EAU development and immune-cell responses against the uveitogenic antigen, reduce the proportion of cells expressing interleukin (IL)-10, interferon (IFN)-γ, and IL-17, and increase the proportion of forkhead box P3 (Foxp3)^+^ cells (50). Kai et al. suggested that the AhR is expressed abnormally in different histotypes in human ovarian cancers and that ITE inhibits the proliferation and migration of OVCAR-3 and SKOV-3 cells through an AhR pathway. Unlike TCDD, ITE is hypotoxic or even non-toxic *in vitro* and *in vivo*, so ITE could be developed as a potent immunosuppressant agent for the treatment of immune diseases and ovarian cancer (51, 52).

##### Kynurenine

The tryptophan metabolites kynurenine, FICZ, and kynurenic acid have been described as natural endogenous AhR ligands that mediate immunosuppressive functions. Kynurenine appears to be the most intriguing tryptophan metabolite in several types of cancers. In numerous cell types, most of the tryptophan is metabolized *via* a kynurenine pathway (53, 54). Kynurenine triggers nuclear translocation of the AhR, thereby enabling activation of its target genes. Indoleamine-2,3-dioxygenase (IDO)1, IDO2, and tryptophan-2,3-dioxygenase 2 (TDO-2) (55) have been shown to be the significant rate-limiting enzymes metabolizing tryptophan to kynurenine. The expression of IDO1 and TDO-2 has been shown to be controlled by the AhR. Such enzymatic activity leads to the exhaustion of tryptophan in the local microenvironment, suppression of antigen-specific T-cell responses, and promotion of the differentiation of T regulatory (Treg) cells during tumor development (56). Emerging evidence suggests that increased expression of IDO in many types of cancers is accompanied with immune escape and cancer-associated inflammation in their microenvironment (57). Opitz et al. demonstrated that kynurenine derived from TDO-2-mediated tryptophan metabolites can inhibit antitumor immune responses and promote the survival and motility of tumor cells in an autocrine, AhR-dependent manner (14).

## The AhR and the Tumor Microenvironment

The physiological effects of AhR activation have been suggested to have significant roles in immune modulation and carcinogenesis. The AhR is expressed at high levels and is chronically active in blood tumors (58, 59), such as T-cell leukemia (60) and lymphoma (61), as well as in solid tumors such as glioblastoma (14), ovarian cancer (51), lung cancer (62), liver cancer (63), and head and neck carcinomas (58). Murray et al. suggested that detection of AhR activity in the microenvironment can serve as a potent diagnostic indicator for tumor aggressiveness (64). Depending on the cancer type, two types of results are associated with AhR activity and the prognosis. Saito et al. indicated that, in hormone-dependent breast cancers, AhR activation is associated with attenuated aggressiveness and a better prognosis (65). However, higher AhR activity has been suggested as being correlated with increased aggressiveness and a poor prognosis in non-small-cell lung cancer (66).

### The AhR and Tumor Development

Strong evidence suggests that constitutively high AhR expression and nuclear localization can be observed in invasive tumor tissues and malignant tumor cell lines (67, 68). The AhR may have important roles in various stages of tumorigenesis owing to its involvement in the inflammatory response and cell-cycle progression (64, 69, 70). The underlying MoA of the AhR in cancers was reviewed in detail by Feng and colleagues (17). With regard to abnormal activation of the AhR with exogenous/endogenous stimulation, certain physiological and pathological processes are disturbed: the proliferation and differentiation of cells, apoptosis, extracellular matrix (ECM) remodeling, angiogenesis, metabolism, and survival. In this way, expression of the target genes is not regulated, and malignant tumors are formed.

The AhR has been suggested to affect cell proliferation in different tumor models and cancer cell lines. In the Hepa1c1c7 cell line, an AhR-defective variant showed delayed progression through the G1 phase in comparison with a wild-type counterpart (71). Studies in a human adenocarcinoma (A549) cell line revealed that DNA binding with the AhR was necessary for the cell cycle and that interaction with an AhR agonist could transform the AhR to its DNA-binding form that stimulated the growth of cancer cells (72). Another study using flow cytometry found that, in AhR-overexpressing cancer cells, 10% were in the S phase and none were in the G2/M phase and that increased expression of transcription factors, replication factors as well as proliferation of cell-nucleus antigens was observed. These results suggested that the AhR can promote proliferation of malignant tumor cells (73). The AhR can also induce cell-cycle arrest. TCDD shows its suppressive effects on gastric cancer cells, breast cancer cells, and retinoblastoma cells to induce growth arrest at the G1-S phase, which can be modulated by persistent phosphorylation of the retinoblastoma tumor suppressor protein *via* cluster of differentiation (CD) K4/6 complexes (74, 75).

Tissue invasion and metastasis are hallmarks of aggressive malignancies. Loss of cell–cell contact triggers the progression and promotion of tumor cells. Increased expression of the AhR is associated with deregulation of cell–cell contact and tumor malignancy. For example, after exposure to the xenobiotic ligand TCDD, cell–cell contact is destroyed, and cell migration and epithelial–mesenchymal transformation (EMT) induced *via* a c-Jun N-terminal kinase-dependent pathway and loss of E-cadherin expression (13). Owens et al. suggested that dissociation of sarcoma (Src) kinase from the AhR complex disrupts cadherin-dependent cell–cell contact (76). Hence, the AhR can reduce cell–cell contact and adhesion and increase the motility and invasiveness of cancer cells, which finally results in the invasion and metastasis of cancer cells.

Another key element involved in the pathogenesis and metastasis of tumor cells is the ECM. Studies have shown that ECM remodulation-associated proteolytic enzymes such as cathepsins (77), urokinase plasminogen activator (uPA) (78), and matrix metalloproteinases (MMPs) (79) are intriguing components of an AhR pathway. Son and Rozman showed, in mouse hepatoma cells, that AhR activation by ligand binding induced expression of uPA protease (80). Similar results have been demonstrated in the studies of Villano and colleagues (81) and Haque and coworkers (82), suggesting that activation of an AhR pathway can enhance MMP expression and result in tumor invasiveness.

Angiogenesis is the physiological process through which new blood vessels form from pre-existing vessels. Angiogenesis and the provision of nutrients and oxygen to support the proliferation of cancer cells also have roles in the aggressiveness and metastasis of tumor cells. During this period, AhR–ARNT heterodimers interact with hypoxia-inducible factor (HIF)-1α to counteract oxygen deprivation and simultaneously upregulate the expression of HIF-1, IL-8, and vascular endothelial growth factor (VEGF) and downregulate expression of transforming growth factor (TGF)-β (83, 84). Angiogenesis is impaired in AhR^(−/−)^ endothelial cells and in AhR-null mice as shown in experiments involving aortic rings. However, this situation can be rescued by VEGF addition. Furthermore, addition of anti-VEGF or knocking out of VEGF in AHR^(+/+)^ cell types results in a reduction of angiogenesis. Experiments on TGF-β in stroma cells elicit the opposite result. The data mentioned earlier suggest that the AhR could be an intriguing regulator and potential therapeutic target for angiogenesis and metastasis during tumor development.

Tumor suppressors regulate the orientation of tumor cells to proliferation or to senescence and apoptosis. Among numerous tumor suppressors, p53 protein shows an obvious interaction with the AhR according to experiments *in vitro and in vivo* (85, 86). In the HepG2 cell line, exposure to TCDD and hypoxia result in inhibition of p53 expression and activation *via* a pathway involving estrogen receptor (ER)α and human double minute-2 and, finally, promotion of tumor progression (67, 87). Furthermore, the AhR has also been reported to affect cancer stem cells and crosstalk with an ER- and inflammatory factor-associated signaling pathway in the pathologic phase of carcinogenesis (88, 89).

Inflammation is also a common feature of tumors. Studies have shown an interaction between AhR activation and expression of inflammatory signaling molecules such as IL-6, IL-10, TGF-β, VEGF-A, signal transducer and activator of transcription (STAT) 6, and nuclear factor-kappa B (NF-κB) (90–93). Kolasa and colleagues found, in a human breast cancer cell (MCF-7) line, that simultaneous exposure to environmental PAHs and tumor necrosis factor (TNF)-α induced increased expression of IL-6 and that this effect could be counteracted by silencing the AhR, implying that AhR may have a key role in IL-6 regulation within the tumor microenvironment (93). The MoA was suggested to be driven by occupancy of AhR–ARNT complexes in DREs, which mediated displacement of histone deacetylase-1 with the IL-6 promoter and subsequently acetylated NF-κB. Dinatale and colleagues presented similar results in head and neck squamous cell carcinoma (HNSCC) lines (58). In the presence of lipopolysaccharide (LPS) in bone marrow dendritic cells (BMDCs), secretion of IL-6, IL-10, and IL-22 has been shown to be regulated through AhR activation (94). Furthermore, the AhR has been shown to bind to NF-κB subunit RelB and that interaction of RelB and AhR in the breast cancer cell lines MCF-7 and MDA-MB-436 induced IL-8 expression (95). John and coworkers observed, in an inflammatory environment in HNSCC lines, that the AhR was likely to regulate the expression or function of several growth factors directly (96). The research mentioned earlier suggests that AhR activation may contribute to inflammatory signaling within a tumor microenvironment through multiple MoAs.

The AhR has been suggested to be a promoter for the initiation and progression of tumor cells, but this view is controversial. Several studies have demonstrated that the AhR may be a tumor suppressor under certain circumstances (74, 97). Wang et al. reported that ITE inhibited the proliferation and migration of ovarian cancer cells *in vitro* and in mice models through the AhR pathway. They also found that TCDD could suppress the proliferation of cancer cells in an AhR-dependent manner at a certain dose (51). Iida and colleagues suggested that N-nitrosobutyl(4-hydroxybutyl)amine suppressed the AhR signaling pathway and finally induced bladder cancer (98).

### The AhR and the Immune System

The AhR has been reported to take part in the modulation of innate immunity and adaptive immunity, which may be involved in tumorigenesis and tumor immune surveillance.

#### The AhR and Innate Immunity

##### The AhR and NK Cells

Natural killer cells are important components of the innate immune system and contribute substantially to antitumor immune responses. Many aspects of the biology of NK cells have been shown to be tightly linked with immune surveillance (99). Upon activation, NK cells can “wipe out” tumor cells by: (i) recognizing tumor-induced immune-activating ligands on host cells *via* receptor activation; (ii) responding to tumor cells without the major histocompatibility complex or other immune-suppressive ligands; (iii) activating cytokines secreted by tumor cells or tumor cell-stimulated immune cells; and (iv) interacting with tumor-infiltrating immune cells such as DCs and macrophages (100).

Increasingly, the AhR has been shown to regulate subsets of immune cells with regard to differentiation and activation *via* cytokine stimulation. However, studies focusing on the relationship between the AhR and NK cells are scarce. Emerging evidence supports a role for NK cells in tumor surveillance (101). Shin and colleagues showed, using *in vivo* experiments, that the cytolytic activity and capacity to suppress formation of RMA-S tumors of NK cells is impaired in the absence of the AhR. AhR activation with the endogenous ligand FICZ can potentiate NK cells to increase IFN-γ secretion and simultaneously enhance cytolytic activity and antitumor activity in an NK cell-dependent manner (100). Wagage and colleagues suggested that AhR activation in NK cells is required for IL-10 production. They isolated NK cells from *Toxoplasma gondii*-infected AhR^(−/−)^ mice and found that IL-10 secretion was impaired and associated with increased resistance to such infection (32). Zhang et al. demonstrated that liver-resident NK cells expressed the AhR constitutively and that in AhR^(−/−)^ mice *in vivo*, deficiency of the AhR in NK cells resulted in increased susceptibility to cytokine-induced cell death (102). These data suggest that the AhR may affect NK cells *via* inflammatory signaling pathways to induce tumor development and immune surveillance.

##### The AhR and Macrophages

Macrophages induce innate immune responses to pathogens through toll-like receptors. Tumor-associated macrophages (TAMs) are critical components of the tumor microenvironment. Masuda et al. demonstrated that interaction between the AhR and STAT1 negatively regulated IL-6 production by inhibiting NF-κB activation and that AhR–specific protein 1 complexes suppress histamine production in macrophages upon LPS stimulation (28). Climaco-Arvizu et al. showed that the AhR can affect the balance between the inflammatory M1 phenotype and anti-inflammatory M2 phenotype by comparing AhR-null mice with wild-type mice: the AhR gene altered macrophage polarization. Activated M2 macrophages have been regarded as being pro-tumor phenotypes in many tumor types (103). Yeung et al. suggested that M2 macrophages contributed to a poor prognosis in hepatocellular carcinoma (HCC) and promoted tumor invasiveness through C–C motif chemokine 22-induced EMT (104). Partecke et al. showed M2 macrophages to be promoters of tumor growth in pancreatic cancer. C57BL/6 mice were injected orthotopically with murine pancreatic cancer cells (6606PDA), and macrophages were depleted by clodronate liposomes. Treatment with M2 macrophages induced tumor growth (105). Zhang et al. investigated the role of M2 macrophages in the progression of colon cancer and found that M2 macrophage-conditioned medium induced the migration of SW480 cells and CD47 expression (106). However, the role of the AhR in TAMs has not been explored. Taken together, these data suggest that the AhR affects tumor development and immune responses within tumor environments *via* TAMs.

##### The AhR and DCs

Considerable attention has been paid recently to the immunoregulatory role of the AhR in DCs. Nguyen et al. showed that LPS and CpG oligonucleotides stimulated BMDCs to express the AhR. AhR^(−/−)^ mature BMDCs induced immune responses with a reduction in expression of kynurenine and IL-10 by treatment with LPS or CpG compared with mature wild-type BMDCs. Upon coculture with BMDCs and naïve T cells, differentiation from naïve T cells to Treg cells was found to be inhibited in AhR^(−/−)^ mature BMDCs. Treatment with l-kynurenine to the system stated earlier rescued this situation. Nguyen et al. concluded that the AhR regulated DC immunogenicity negatively *via* a kynurenine-dependent MoA (107). Thatcher et al. demonstrated that AhR-knockout mice developed intense allergic responses to the allergen ovalbumin with increased activation of DCs. Deficiency of the AhR resulted in enhanced activation of T cells by pulmonary DCs and intense pro-inflammatory allergic responses (108). Wang and colleagues explored the influence of AhR activation by ITE and FICZ on the differentiation, maturation, and function of monocyte-derived DCs (MODCs) in patients with Behçet’s disease. AhR activation by FICZ or ITE inhibited DC biology with reduced production of TNF-α, IL-1β, IL-6, and IL-23 and increased secretion of IL-10 (109). Vogel et al. showed that the AhR modified maturation of BMDCs accompanied with increased expression of IDO and altered secretion of cytokines, chemokines, and DC-specific surface markers and receptors (110). Kado et al. demonstrated that the AhR modulated toll-like receptor-induced expression of cytokines and DC-specific surface markers in human MODCs involving NF-κB RelB and the immune regulatory factor caudal type homeobox-2 by treatment with ligands, such as TCDD, FICZ, and I3C, but not kynurenine (111). Taken together, these studies suggest that AhR activation through exogenous or endogenous ligands affects the function and differentiation of DCs with regard to maintaining immune homeostasis.

Dendritic cells also act as antigen-presenting cells in terms of initiating adaptive immune responses, including the differentiation and polarization of T cells and B cells. Jurado-Manzano et al. found that FICZ activated the AhR in MODCs, promoted the differentiation and maturation of DCs, and induced naïve T cells to differentiate into CD4^+^CD25^+^Foxp3^+^ Treg-like cells to cause immune tolerance (112). Ping and colleagues showed, in allergic rhinitis (AR) patients, that the AhR modulated the increased secretion of IL-10 in DCs and CD4^+^ T cells, reduced expression of IL-1β and IL-6 in DCs and IL-17 in CD4^+^ T cells, *via* ITE treatment, and subsequently inhibited the response of T-helper (Th)17 cells to suppress the AR (113). De Araújo et al. demonstrated the fundamental role of the IDO–AhR axis in adjusting the balance between Th17 cells and Treg cells in pulmonary paracoccidioidomycosis through its effects on plasmacytoid DCs (114). The research results mentioned earlier were confined to inflammatory or autoimmune diseases. However, there are very few reports on the relationship between the AhR and DCs in tumors, and further investigations are warranted.

#### The AhR and Adaptive Immunity

An adaptive immune response is triggered *via* activation, differentiation, and clonal expansion of lymphoid lineage cells (T and B cells). Studies have shown that tumors “escape” from immune surveillance *via* inactivation or deletion of self-reactive T cells and B cells, which is an important early event in tumor development (114, 115).

##### AhR and T Cells

Type-1 regulatory T cells (Tr1), thymus-derived Treg cells, and Th17 cells have central roles in mediating immunosuppressive effects within the tumor microenvironment by suppressing the proliferation and cytokine secretion of effector cells (116). The AhR regulates CD4^+^ T-cell differentiation, and thus, AhR levels are increased in this process (117, 118). The AhR can induce Treg cells and Th17 cells based on the TCDD concentration in EAE, suggesting that the balance between Treg cells and Th17 cells has a key role in autoimmune disease (8). Considering the immunosuppressive effects of AhR ligands on autoimmune disease, it is rational to propose that AhR activation in the tumor microenvironment is associated with an increased proportion of Treg cells and may explain (at least in part) the tumor-promoting properties of TCDD.

On account of the phenomena observed in models of autoimmune disease, one could infer that TDO-derived kynurenine induces the differentiation of Treg cells and suppresses tumor-specific CD8^+^ T cells. For instance, Opitz et al. suggested that kynurenine affected the proliferation of CD4^+^ and CD8^+^ T cells in a concentration-dependent manner and verified that kynurenine suppressed antitumor immune responses through the AhR in sections of human gliomas (14, 119). Presence of the AhR is necessary in T cells for optimal generation of Foxp3^+^ Treg cells and kynurenine induces generation of Foxp3^+^ Treg cells in an AhR-dependent manner (30). The AhR is also crucial for the formation of Tr1 cells in mice and humans, which inhibit autoimmune responses by interaction with the transcriptional factor macrophage-activating factor to enhance expression of IL-10, IL-21, and IL-27 (120). AhR activation is also involved in the promotion of Th17 to Tr1 transdifferentiation (98).

Whether the AhR can modulate antigen-specific CD8^+^ T-cell responses is controversial. Winans et al. demonstrated that AhR deficiency affected primary CD8^+^ T-cell responses with altered patterns of DNA methylation in a cell-extrinsic manner during infection with an influenza virus (121). The role of the AhR in tumor immunity has not been explored in depth and merits further investigation.

The AhR can regulate the apoptosis of process of T cells *via* modulation of expression of Fas and Fas ligand by exposure to TCDD or other endogenous ligands. Several key pathway molecules in the tryptophan pathway, including IDO-1, TDO-2, and kynurenine, participate in controlling immune tolerance and promoting tumor escape by regulating T cells and the proliferation, differentiation, and apoptosis of tumor cells *via* the AhR (122). However, the underlying molecular MoA is incompletely understood, and deeper investigations are needed.

##### The AhR and B Cells

Recent data have suggested the potential role of the AhR in regulation of the function of B cells involved in the tumor microenvironment (123, 124). The AhR has been reported to mediate B-cell differentiation from hematopoietic stem cells into pro-B cells, mature B cells, and plasma cells (61). For example, B cells are sensitive targets for TCDD. In LPS-activated CH12LX B lymphoma cells with simultaneous TCDD treatment, the DNA binding and transcriptional activity of activated protein-1 and immunoglobulin expression were repressed markedly, which revealed possible associations between the AhR and the genes pivotal to the maturation and function of B cells (123). More than 1,000 human cancer cell lines generated at the Broad Institute of the Massachusetts Institute of Technology and Harvard University have undergone microarray analyses. Data revealed that myelomas and B-cell lymphomas (diffuse large phenotype and unspecified phenotype) showed reduced expression of the AhR, but notably increased expression in chronic lymphocytic leukemias and Hodgkin’s lymphomas (61). Interaction between the AhR and TCDD and other ligands contribute to cancers derived from B-cell lineages by affecting the growth and survival of cells *via* the tumor microenvironment. Further studies on the function of the AhR in B cell-associated microenvironment in solid tumors must be explored.

## The AhR and Solid Tumors

### The AhR and Malignant Gliomas

Emerging evidence has demonstrated the role of the AhR and its ligands in brain tumors. The generation of reactive oxygen species may have a role in the underlying MoA of AhR-mediated glioma, as well as the activation of glutamate receptors, histone acetylation, signal transducers, peroxisome proliferator-activated receptors, and transcription activators (125). Gramatzki et al. explored AhR expression and its MoA in human glioma cells and found that AhR inhibition downregulated expression of the TGF-β/Smad [mothers against decapentaplegic homolog 1 (Drosophila)] pathway (59). Silginer and colleagues identified a signaling network composed of the AhR, integrins, and TGF-β. They showed integrin inhibition to be a prospective strategy to tumor inhibit angiogenesis and to restrain the AhR- and TGF-β-controlled characteristics of malignancy in glioblastomas (126). Dever and Opanashuk explored the function of the AhR in a medulloblastoma cell line (DAOY). They suggested that abnormal activation or suppression of the AhR could dysregulate the cycle of granule neuron precursor (GNP) cells and that the AhR could promote the proliferation of medulloblastoma cells (127). Adams et al. provided a new perspective on how dysregulation of the kynurenine pathway affects antitumor immune responses. Tryptophan metabolites such as 3-hydroxyanthranilic acid, quinolinic acid, and kynurenine as well as regulatory enzymes such as IDO-1 and TDO-2 have central roles in antitumor immunity and are dependent on the AhR signaling pathway (128). Bostian et al. demonstrated that activation of the AhR pathway *via* TDO-2 increased expression of kynurenine and human Y-family polymerase κ, which resulted in genomic instability and high levels of replication stress in glioblastomas (129). Opitz et al. found that the TDO–kynurenine–AhR pathway is closely associated with malignant progression and a poor prognosis. They found antitumor immune responses to be suppressed and the proliferation and survival of tumor cells to be promoted by TDO-derived kynurenine in an autocrine/paracrine AhR-mediated manner (14). Taken together, these data suggest that the AhR may affect the growth of brain tumors and antitumor immune responses.

### The AhR and Breast Cancer

Constitutive activation of the AhR has been examined in different models of breast cancer in mice and humans (75, 130, 131). The AhR is involved in several cell-signaling pathways, including interaction with cytokines, tyrosine kinases, and growth factors. Belguise and colleagues and Vogel et al. suggested a connection between AhR activity and upregulation of the transcriptional genes associated with the invasion and survival of cancer cells (132, 133). Goode and coworkers and Parks et al. demonstrated that knockdown or inhibition of the AhR led to downregulation of expression of tumor cells and metastasis-associated genes, as well as inhibition of the invasion and migration of cancer cells (130, 134). In addition, hyperactivation of the AhR with exogenous and endogenous ligands may induce different signaling pathways and lead to reduced invasion of breast cancer cells (70, 135, 136). Abnormal tryptophan metabolism and increased production of tryptophan metabolites have also been documented in human breast cancers. D’Amato et al. demonstrated that a TDO2–AhR signaling axis in a kynurenine pathway promoted anoikis resistance *via* NF-κB and highlighted that TDO-2 could be an intriguing target for the treatment of triple-negative breast cancer (137). Bekki et al. explored the anti-apoptotic function of the AhR in breast cancer cells by exposure to ultraviolet light, TCDD, and kynurenine, respectively. They suggested that TCDD and kynurenine mediate tumor immunity *via* the suppression of apoptosis, accompanied with the induction of expression of the COX-2 and NF-κB subunit RelB (138). Saito and colleagues demonstrated the AhR to be a newly defined prognostic factor for breast cancers. They showed that AhR^+^ breast cancer patients had a relatively better prognosis than those with AhR^−^ breast cancer because of the effects of activating AhR on cell proliferation and expression of MMPs genes (65). Most of these studies were confined to the tumor itself and more attention should be paid to role of the AhR in the microenvironment of breast cancers.

### The AhR and Lung Cancer

Exposure to cigarette smoke and environmental pollutants is the dominant pathogenesis for lung cancer. PAHs are exogenous ligands that bind to the AhR, which reacts with PAHs *via* Phase I CYP enzymes to sequentially influence the initiation, promotion, and progression of lung cancer. Oyama et al. tested 78 non-small-cell lung cancer samples and observed a direct correlation between expression of the AhR and downstream expression of CYP1a1, most notably in adenocarcinomas (139). Lin et al. also reported that lung adenocarcinoma tissues and cell lines expressed increased AhR at mRNA and protein levels (140). Gao et al. found AhR overexpression to be associated with an increase in nuclear translocation of RelA, the AhR–RelA complex, and NF-κB activity, giving rise to upregulation of IL-6 secretion (which is critical for lung cancer initiation) (141). Lung carcinogenesis has also been hypothesized to be *via* crosstalk with nuclear factor (erythroid-derived 2)-like 2 and ER, thereby providing effective targets for the AhR to prevent and treat lung cancer.

### The AhR and Liver Cancer

The AhR conduces the regulation of the communications, adhesion, migration, and proliferation of cells in liver carcinogenesis. Fan et al. showed that AhR activation led to arrest of the G0–G1 phase in the cell cycle and diminished the competency for DNA replication and suppression of cell proliferation. They also found that the AhR was a cancer-suppressor gene in the absence of a xenobiotic ligand and that its silencing may be linked with cancer progression (97). de Tomaso et al. demonstrated that AhR was a mediator in the extracellular signal-regulated kinase1/2 signaling pathway and contributed to the regulation of the cell cycle in hexachlorobenzene-treated HepG2 cells (142). Andrysik et al. investigated if toxic AhR agonists may synchronously relieve contact inhibition and reduce gap junctional intercellular communication *via* regulation of connexin-43 (143). Terashima et al. suggested that the AhR induced VEGF expression by activation of activating transcription factor 4 during glucose deprivation in the HepG2 cell line, which affected the malignancy of liver cancer (144). Kennedy et al. found that tumor promotion by TCDD was attributed to activation of the AhR and TNF/IL-1 receptors in liver cancer (145). Koch et al. observed that flutamide activated the TGF-β1 signaling pathway *via* the AhR and influenced some biologic characteristics in human HCCs (146).

### The AhR and Solid Tumors in Children

Neuroblastoma is the most common malignant solid tumor of infancy. Wu et al. found AhR expression to be correlated negatively with N-myc proto-oncogene (MYCN) expression and highly correlated with the histology grade of differentiation in human neuroblastoma tissues, suggesting that the AhR regulates the expression and function of MYCN upstream through modulation of E2F transcription factor 1 (147). Huang et al. demonstrated that knockdown of micro-RNA-124 promoted the differentiation, cell-cycle arrest, and apoptosis of the neuroblastoma cell line SK-N-SH *via* the AhR signaling pathway (148). Little is known about medulloblastoma and the AhR. Dever and Opanashuk reported that the AhR was overexpressed in the GNP cells from the developing cerebellum and that abnormal activation/suppression of the AhR led to aberrant regulation of their cell cycle and maturation, suggesting that the AhR stimulates the growth of medulloblastomas (127).

### Other Solid Tumors

The AhR is also constitutively active in prostate cancer, melanoma, ovarian cancer, colon cancer, and gastric cancer (51, 149, 150). Xie et al. found that Src-mediated crosstalk between the AhR and epidermal growth factor receptor stimulated proliferation of colon cancer cells (151). Villano et al. investigated how AhR activation affected several melanoma cell lines and normal human melanocytes. They hypothesized that expression of the AhR and ARNT activated by TCDD in the transformed melanoma cell line A2058 resulted in increased expression and enhanced activity of MMP-1, MMP-2, and MMP-9 (81).

## Concluding Remarks and Future Perspectives

**Figure 3:**
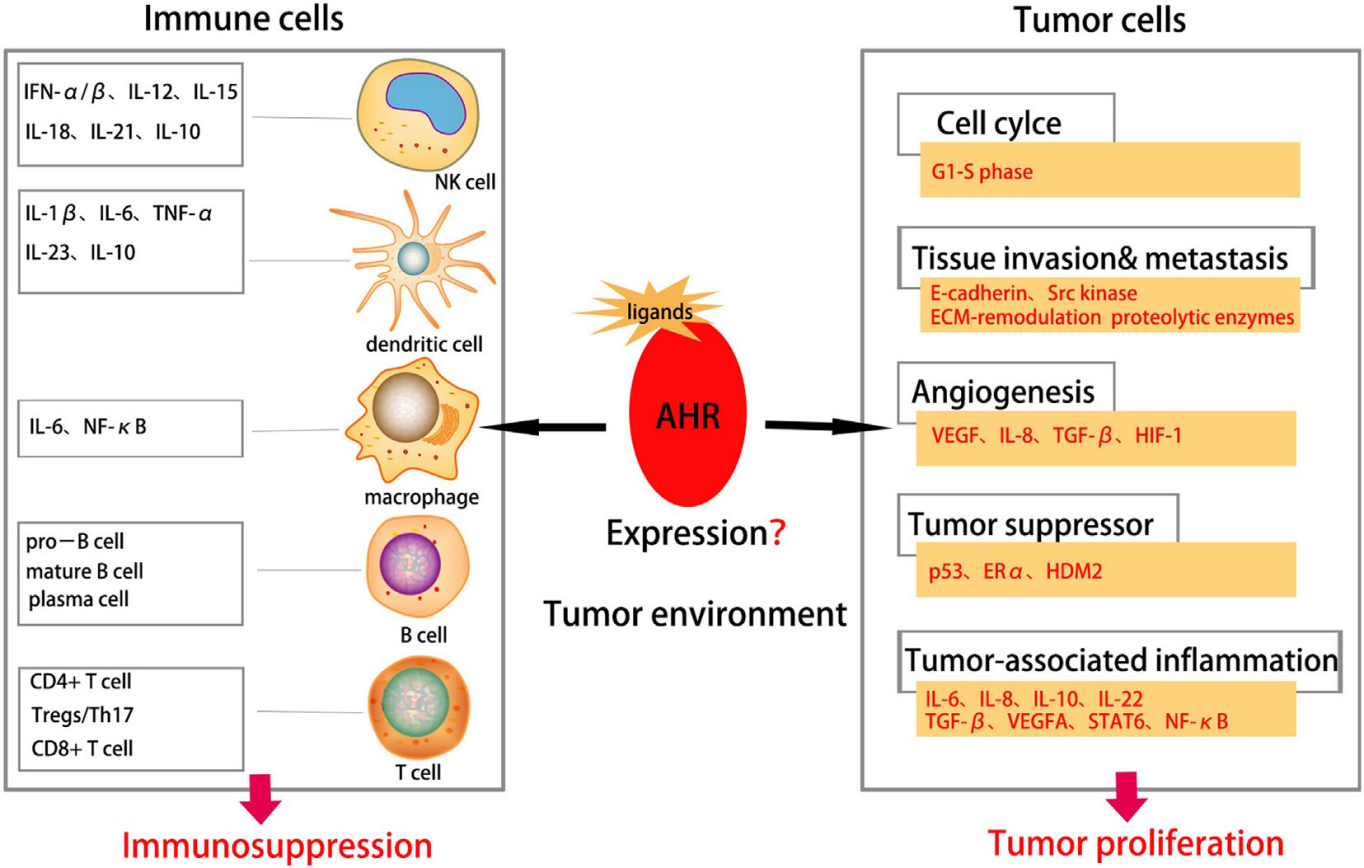
Aryl hydrocarbon receptor (AhR) in tumor environment. Abnormal expression of AhR in tumor environment affects both the immune cells and tumor cells in tumor environment. The AhR plays an important role in various stages of tumorigenesis, including cell proliferation, tissue invasion, angiogenesis, tumor-associated inflammation, metastasis etc., suggesting AhR as a promoter for tumor initiation. It can also suppress proliferation and function of immune cells thus suppressing tumor immune responses.

Overexpression and constitutive activation of the AhR have been observed in various tumor types and is associated with histology grade. The AhR occupies an important place in multiple stages of the development and progression of cancer cells; whether it functions as an oncogene or suppressor gene merits further investigations. Tumor immunity is one of the most important and promising fields in oncology. The AhR is not only a transcription factor responding to toxins but also crucial in the physiological functions of immune-cell compartments. Additional in-depth investigations of AhR function in the tumor microenvironment, including tumor cells and immune cells, and the relationship between immunity and tumors are warranted (Figure 3). Among AhR signaling and other pathways, the kynurenine pathway is a new and prospective way to link the immune system and tumors. The immunosuppressive role of the AhR in tumors suggests that targeting the AhR and associated signaling pathways may provide a novel therapeutic strategy for cancer.

## Author Contributions

PX, the first author, contributed to collection of references and manuscript preparation. JF and YZ contributed to the modification of the manuscript.

## Conflict of Interest Statement

The authors declare that the research was conducted in the absence of any commercial or financial relationships that could be construed as a potential conflict of interest.
